# Domestic Economy in Isolation Hospitals

**Published:** 1916-03-04

**Authors:** 


### THE EDITOR'S LETTER-BOX.

**Our Correspondents are reminded that brevity of style and conciseness of statements greatly facilitate early insertion We cannot in any way be responsible for the opinions expressed by correspondents, who must give their name and address as a guarantee of good faith, but not necessarily for publication.**

### Domestic Economy in Isolation Hospitals.

*To the Editor of* THE HOSPITAL.

SIR,—"Another Isolation Matron" is not alone in her dislike to estimates reckoned on a percentage basis. It is easy to simplify percentages of additional cost by altering them to the number of shillings in a pound, and this may be done in a moment by dividing by five. For instance, 25 per cent. is 5s. in the £, 15 per cent. is 3s. in the £, and so on. When it is estimated that prices have gone up 30 per cent., it means that for every pound's worth of goods as purchased a year or two ago an extra 6s. has now to be paid. This is concrete, and its bearing on the weekly books can be grasped by all who have to use the articles in question. In some hospitals excellent results have been obtained by circulating in each ward lists showing the increased cost of dressings and ward necessaries. It would stimulate economy in the kitchen if the same plan were pursued with the food. THE WRITER OF THE ARTICLE.

